# Microstructural Characteristics and Properties of Laser-Welded Diamond Saw Blade with 30CrMo Steel

**DOI:** 10.3390/ma17081840

**Published:** 2024-04-17

**Authors:** Qiang Xu, Chen Shu, Yibo Liu, Shengzhong Kou, Rui Cao, Xiaodie Cao, Jiajun Wu

**Affiliations:** 1 Central Iron & Research Institute, Beijing 100081, China; xuqiang@gangyan-diamond.com (Q.X.); shuchen0518@163.com (C.S.); liuyibo@gangyan-diamond.com (Y.L.); 2 Beijing Gang Yan Diamond Products Company, Beijing 102200, China; 3 School of Material Science and Engineering, Lanzhou University of Technology, Lanzhou 730050, China; koushengzhong@163.com (S.K.); caorui@lut.edu.cn (R.C.); 4 College of Engineering, Shantou University, Shantou 515063, China; 23xdcao@stu.edu.cn

**Keywords:** laser welding, laser power, welding speed, mechanical properties, microstructural characteristics

## Abstract

In order to enhance the quality of diamond composite materials, this work employs a Cu-Co-Fe and Ni-Cr-Cu pre-alloyed powder mixture as a transition layer, and utilizes laser-welding technology for saw blade fabrication. By adjusting the laser-welding process parameters, including welding speed and welding power, well-formed welded joints were achieved, and the microstructure and mechanical properties of the welded joints were investigated. The results demonstrate that the best welding performance was achieved at a laser power of 1600 W and a welding speed of 1400 mm/min, with a remarkable tooth engagement strength of up to 819 MPa. The fusion zone can be divided into rich Cu phase and rich Fe phase regions, characterized by coarse grains without apparent preferred orientation. The microstructure of the heat-affected zone primarily consists of high-hardness brittle quenched needle-like martensite, exhibiting a sharp increase in microhardness up to 550 HV. Fracture occurred at the boundary between the fusion zone and the heat-affected zone of the base material, where stress concentration was observed. By adjusting the welding parameters and transition layer materials, the mechanical properties of the joints were improved, thereby achieving a reliable connection between diamond composite materials and the metal substrate.

## 1. Introduction

Diamond, known for its exceptional hardness, wear resistance, high thermal conductivity, and low coefficient of friction [1], finds extensive applications in various industrial fields. It is widely used for the highly efficient cutting of silicon wafers and hard, brittle materials [2], as well as in electronic component packaging [3] and precision tools utilized in aerospace [4]. In recent years, scholars have conducted extensive research on workpieces with special shapes made from metal-matrix diamond composite materials. For instance, Tao et al. [5] successfully prepared Co-Cr-Mo/diamond composites with excellent wear resistance using selective laser melting technology, providing a new method for manufacturing complex diamond tools with high-concentration cutting edges in a refractory metal matrix. Additionally, Hou et al. [6] achieved the successful preparation of metal-based diamond super-hard bits with a uniform element distribution and excellent mechanical properties through the addition of pre-alloy powders (Fe, Co, Cu) and microwave press-free sintering technology.

Efficiently joining the composite part to the matrix is a challenging and crucial task. Various methods have been adopted to achieve effective joining between metal/diamond components and substrates, including integral sintering, brazing, radio frequency welding, vacuum welding, laser welding, and selective laser melting [7,8,9]. However, traditional integral sintering and radio frequency welding processes are not suitable for high-efficiency and high-strength joining requirements. Integral sintering faces complexity in producing metal-based diamond tools with large sizes due to the intricate production process [10,11]. Similarly, the limited welding strength and accuracy of radio frequency welding hinder the manufacturing of cutting tools with high processing efficiency. Moreover, the stress concentration between the metal/diamond component and the matrix can lead to joint failure, causing safety accidents under harsh conditions such as water-free cooling, dry cutting, and dry drilling [12,13,14].

Laser-welding technology offers a promising approach to prepare diamond tools by welding a tool head, containing a metal binder and diamonds sintered through powder metallurgy, onto a steel matrix [15]. Laser-welding diamond saw blades exhibit advantages such as deep penetration, high efficiency, and a large fusion area [16]. These blades are not affected by air pollution and can withstand significant impacts at high temperatures [17]. However, direct welding of diamond segments onto the substrate often results in the presence of numerous pores and cracks, leading to an inadequate joining strength [18]. The effective joining of diamond composite materials and metal substrates using laser welding poses challenges due to the significant thermal mismatch and the formation of brittle intermetallic compounds [19]. One potential solution to address these challenges is the introduction of a transition layer, which provides a gradual change in material properties, reduces thermal stress, and promotes metallurgical bonding between the diamond composite and metal substrate. The addition of a transition layer, typically with a thickness of 1~2 mm, has proven effective for preparing laser-welded saw blades.

Notably, pre-alloyed powder plays an increasingly important role as a transition layer in the preparation of metal-based diamond composites [20]. Li et al. [21] used Co-Cr-Ni powder as a transition layer to join titanium alloy and steel. It was found that the diffusion of Co, Cr, and Ni alloy elements to the interfacial layer in diamond drill head matrix material leads to the formation of infinite solid solution and avoids the formation of a brittle intermetallic compound, which significantly improves the plasticity, toughness, and crack resistance. In order to improve the welding quality of the copper and stainless steel, Xin et al. [22] successfully obtained good welded joints by laser welding with Ni as the transition layer and investigated the microstructure and mechanical properties of the joints. The results demonstrated that the addition of Ni as a transition layer can effectively prevent the formation of welding defects and spherical particles, and improve the mechanical properties of the joints. Li et. al. [23] use Cu as a transition layer and laser-welding technology to join TiNi alloy and stainless steel. It was concluded that, with the increase in copper thickness, the formation of the soft copper solid solution phase and the decrease in brittle intermetallic compounds result in an increase in the tensile properties of welded joints. The transition layer cannot contain low melting point metals which easily evaporate and vaporize. The transition layer can be composed of the single-element constituent containing Co or Ni, the double-element formula containing Fe Co, Fe Ni, Co Ni, Fe Cu, etc., or the three-element constituent containing Fe Co Ni [24,25,26]. In addition, if the densification of the sintered transition layer is not enough, welding pores are easily produced, which leads to a decrease in the welding strength. Hence, the density of the tool head and the hardness of the welding transition layer should be ensured during the manufacturing process of the tool head [27].

The selection of transition layer material for laser-welding diamond cutting heads and metal substrates should consider factors such as thermal conductivity, oxidation susceptibility, brittleness, corrosion resistance, and compatibility with both diamond and metal [28]. Due to the high cost and strategic resources, a mixture and pre-alloyed powder as a transition layer of cobalt powder with iron and nickel powder tends to be used in laser welding. In addition, the inclusion of copper improves formability and mass density. Therefore, in this study, Cu Co Fe and Ni Cr Cu pre-alloy powders were mixed as a transition layer aiming to join the diamond segments and 30CrMo matrix by laser welding. The microstructure and properties of metal-based diamond composites can be controlled by adjusting the laser-welding parameters, such as welding speed and welding power. By characterization of the microhardness, relative density, and bending strength, the optimal laser-welding parameters were selected. 

## 2. Materials and Methods

### 2.1. Preparation of Experimental Sample

**Figure 1:**
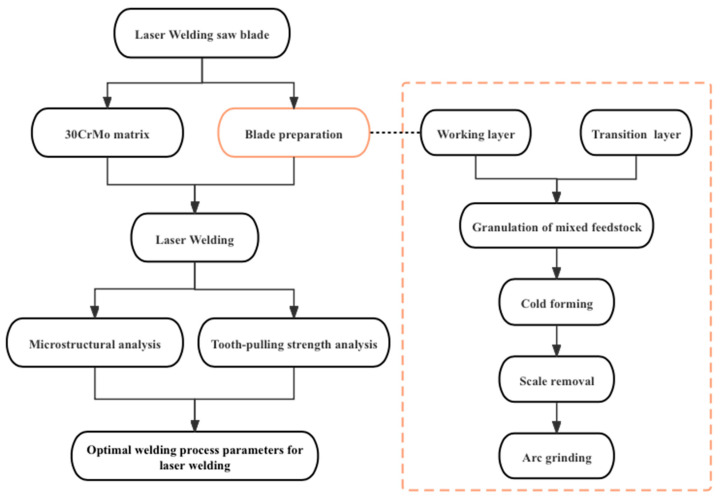
Flowchart for experimental research.

The main flow of this study is shown in Figure 1; in this experiment, 4 to 5 sets of saw blades were prepared based on different welding process parameters and a tooth-breaking test was conducted to obtain experimental results. Considering that the preparation of metallographic samples requires a set of saw blades, while the calculation of tooth-breaking strength and the examination of post-tooth-breaking morphology necessitate three sets of saw blades. To ensure the repeatability and accuracy of the research findings, multiple sets of saw blades were prepared during practical operations, taking into account the potential occurrence of issues during sample preparation. The diamond and pre-alloy powder in the working layer were mixed for 2~3 h in the 3D mixture machine. Then, the spherical particles were prepared with a granulating agent with 5% wt by sifting method. 

Moreover, the pre-alloy powder in the transition layer was mixed and granulated in the same way, and the chemical component of the transition layer is shown in Table 1. The cold-pressed green body containing both the transition layer and working layer, which was sintered and formed in the hot-pressing sintering machine, was obtained by using a four-column hydraulic press. Prior to this experiment, we conducted a systematic study on the organizational structure and mechanical properties of the sintered carcasses of the cutterhead with different sintering process parameters, focusing on hardness, densification, and flexural strength, so as to determine the optimal sintering temperature and pressure. The sintering temperature and pressure were 800~900 °C and 25 MPa, respectively. A segment with a size of 10 mm × 2.6 mm × 38 mm and a transition layer thickness of 2 mm was prepared. The welding process was employed upon the actual diamond saw blade, with the widths of the blade segments and the matrix being predetermined. The laser-welding process was conducted in ambient air without the utilization of any shielding gas for protection. Additionally, the blade segments possessed a thickness of 2 mm, while the matrix exhibited a thickness of 1.8 mm. To ensure uniformity, the segments were processed by arc grinding, aligning the curvature of the welding position with that of the 30CrMo steel matrix. Subsequently, the segments were welded to the matrix using different welding parameters, considering the external diameter of 210 mm and a thickness of 1.8 mm. It is worth noting that the chemical component of 30CrMo is shown in Table 2.

**Figure 2:**
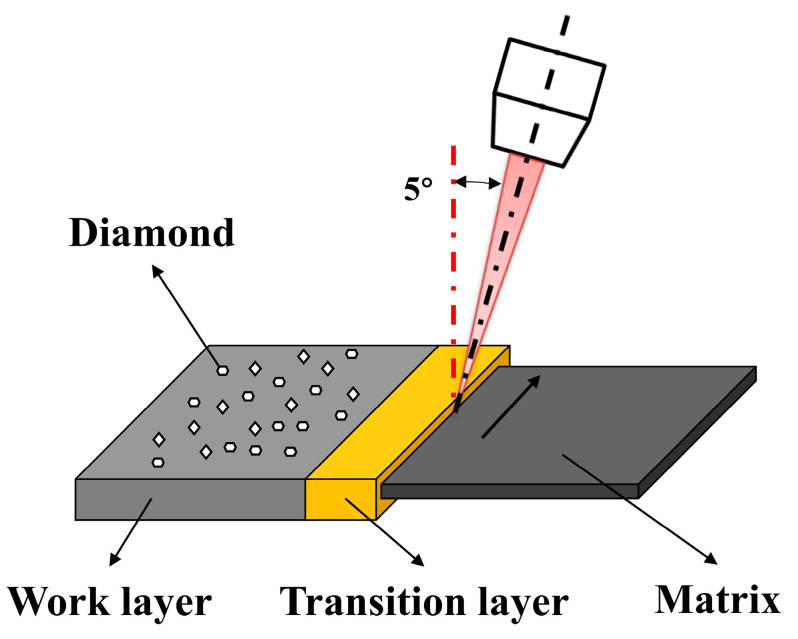
Schematic diagram of laser-welded diamond saw blade.

The laser welding was performed by an automatic laser welder (LSM240, Dr. Fritsch, Fellbach, Germany) with a maximum laser power of 2.5 kW and laser mode TEM00. Focus position parameters of laser welding remained unchanged (defocus: −1, spot ratio: 3.7, incident angle: 5°). The welding schematic diagram is shown in Figure 2. The critical factors in controlling the microstructure and properties of the composite material during laser welding include the composition of the material, laser power density, welding speed, and beam size. Adjustments in welding parameters such as speed and power can significantly influence the outcomes by affecting the heat input, cooling rate, and solidification behavior, thereby influencing the microstructural features, mechanical properties, and defect formation in the composite material. Therefore, the optimization of welding process parameter tests was carried out by changing the welding power and welding speed. The welding parameters are shown in Table 3. 

### 2.2. Material Characterization

After laser welding, the metallographic specimens were cut by electrical discharge machining (EDM), and then were ground, polished, and etched. Various etching solutions were used to clearly show the microstructure of various regions of the whole laser-welded joint. The steel substrate and heat-affected zone were etched by a 3% (mass fraction) solution of nitric acid in alcohol. The transition layer was etched by a solution of FeCl_3_·HCl. The molten zone between the blade and substrate was etched using aqua regia (hydrochloric acid: nitric acid = 1:3) solution. After etching, the metallographic microstructure of the specimens was observed and analyzed by an Ultra-Depth Three-Dimensional Microscope (VHX-2000, KEYENCE, Osaka, Japan) and scanning electron microscopy (JSM-6380LV, JEOL, Showa, Japan). The microhardness of the joint was measured using a digital superficial Rockwell hardness tester (HRMS-45, Gelaima, Suzhou, China) with a 30 kg load and superficial Rockwell scales of HR30T. The average of five sample measurements was recorded as the result.

The phase of each area for the welded joint was comprehensively analyzed by XRD (X-ray diffraction) and EDS (energy dispersive spectroscope). Additionally, grain size and grain orientation were analyzed by EBSD (electron backscatter diffraction) analysis [29]. The weld metal’s tooth-pulling strength was measured, which can be expressed as follows.

(1) $$M_{b}=\sigma_{b}\cdot L\cdot E^{2}/6$$

where the tooth-pulling torque, denoted as *M_b_* in Formula (1), is determined by the length of the interface between the blade and the substrate, which is generally equal to the tooth length (*L*). *σ_b_* is defined as tooth-pulling strength. Additionally, *E* represents the thickness of the substrate wall, while 6 signifies its bending strength [30].

## 3. Results and Discussions

### 3.1. Effect of Laser Power on Weld Formation and Tooth-Pulling Strength

Laser power and its corresponding energy density play a key role in welding processes [31,32]. An optimized laser power and its corresponding energy density can be obtained and regarded as the critical threshold. The penetration depth becomes lower when the power density is lower than the critical threshold. Once the power density reaches or exceeds the threshold, the penetration depth significantly increases. When the power density and weld depth are less than 10^6^ W/cm^2^ and 0.5 mm, respectively, the welding processes remain in a stable heat conduction mode, with only melting occurring on the surface of the workpiece. When the power density exceeds 10^6^ W/cm^2^, the metal is rapidly vaporized, a spoon-shaped deep hole and plasma are formed, and laser-deep penetration welding is achieved. While a power density of 10^6^ W/cm^2^ may be sufficient to achieve deep penetration welding in this work, it is not a universal threshold. The specific power density required for deep penetration welding depends on multiple factors, including the material, laser parameters, and welding conditions.

**Figure 3:**
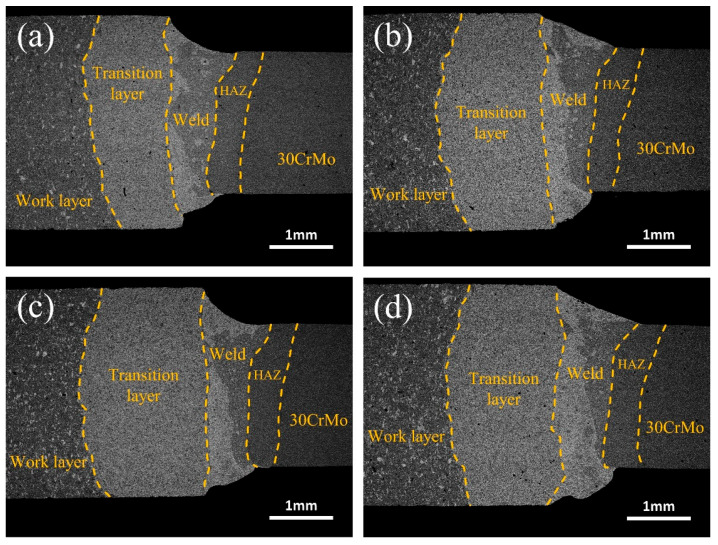
Effect of laser power on the weld appearance between the diamond saw blade tip and the substrate: (**a**) 1400 W; (**b**) 1500 W; (**c**) 1600 W; (**d**) 1700 W.

Figure 3 shows the weld appearance between the diamond saw blade head and the substrate at different laser powers. From Figure 3, it can be seen that, as the laser power increases, the volume of the cup-shaped molten pool gradually expands, showing a shape that is wide at the top and narrow at the bottom. The top of the welded joint is a relatively smooth curved surface. The high-power laser energy leads to an increase in the melting volume of Fe in the substrate and Cu, Ni, etc. in the transition layer, which expands the molten pool to a larger area. In addition, the material on the edge of the weld pool melts and flows faster under the high-power laser energy, which makes a distinct protrusion at the bottom of the weld joint. Upon conducting XRD scans on the transition layer, consistent fluctuations in composition were observed, while no discernible alterations in the microstructure or significant changes in grain size were detected. This accounts for the absence of a pronounced thermal-affected zone on the left side. 

**Figure 4:**
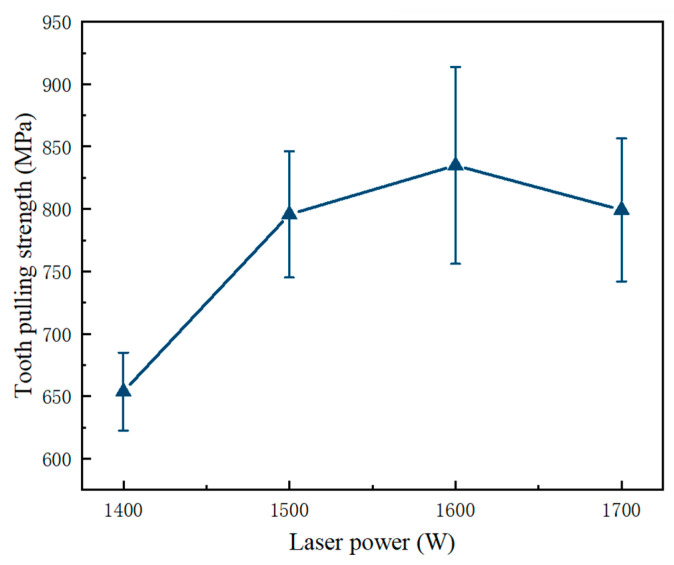
Effect of laser power on the tooth-pulling strength of the welding joint between the diamond saw blade head and the substrate.

During the high-speed cutting process of diamond saw blades, welding strength is a crucial parameter for their safety. Typically, the welding strength is characterized by destructive bending strength (tooth-pulling strength) [33,34]. Figure 4 illustrates the effects of laser power on the tooth-pulling strength of the welded joint between the diamond saw blade head and substrate. At a laser power of 1600 W, the tooth-pulling strength reaches its maximum value of 819 MPa. The average tooth strength of 819 MPa is achieved under the welding process characterized by a laser power of 1600 W and a welding speed of 1400 mm/min. In the absence of any variations in the composition of the transition layer or sintering process parameters, or the dimensions of the cutting tool and substrate, it is deduced that the welding process parameters singularly influence the tooth strength. As the laser power increases from 1400 W to 1600 W, there is a gradual improvement in tooth-pulling strength.

Higher laser power can provide adequate thermal energy to rapidly melt the welding material and form a uniform molten pool. This homogeneous molten pool effectively eliminates defects and impurities in the welded joint, thereby providing a superior bonding strength. However, when the laser power exceeds 1700 W, the welding strength shows a significant reduction due to an increase in defects such as porosity and cracks. Additionally, excessive laser power can lead to excessive melting within the welded joint, resulting in changes in its microstructure, grain growth, and crack formation. Additionally, high-power input can lead to an increase in thermal deformation and residual stress, which further decreases the tooth-pulling strength of the welded joint. The quality of laser welding is typically assessed by evaluating criteria like weld strength, weld geometry, and absence of defects, and conducting non-destructive testing methods, such as visual inspection, X-ray, or ultrasonic examination. The laser-welding process involves focusing a high-intensity laser beam on the workpiece, melting and solidifying the material to create a strong weld joint, offering advantages like precision, speed, and a minimal heat-affected zone compared to traditional welding techniques.

### 3.2. Effect of Welding Speed on Weld Formation and Tooth-Pulling Strength

In laser-deep penetration welding, the welding speed plays a crucial role in determining both the welding depth and the tooth-pulling strength when the laser power stays constant [18,35]. A higher welding speed leads to shallower melting, hindering the quick escaping of gas from the molten pool and consequently generating numerous pores, which decrease tooth-pulling strength. Conversely, a slower welding speed can cause excessive melting of the transition layer, expanding the heat-affected area and coarsening the microstructure. When adequate depth can be ensured, the choice of a higher welding speed can increase production efficiency and reduce costs. Moreover, lower welding speeds can lead to excessive heat input, and deterioration in the microstructure and performance of the weld, and even the appearance of macroscopic cracks.

**Figure 5:**
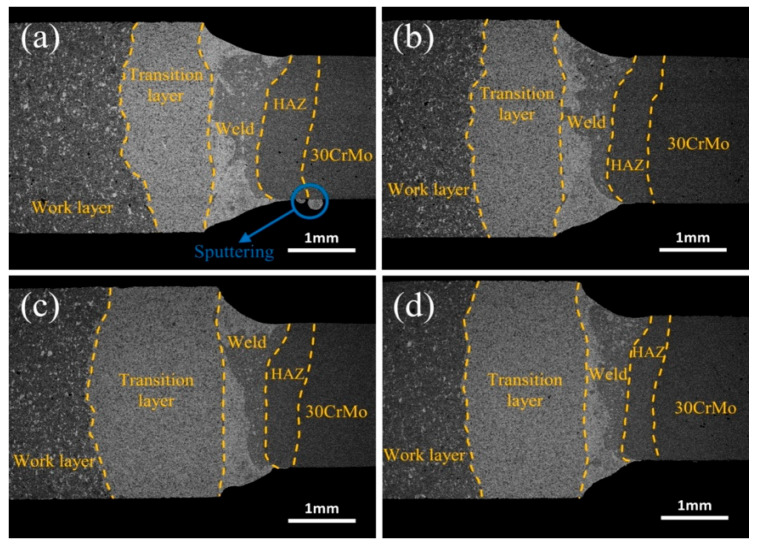
Effect of welding speeds on the weld morphology between diamond saw blade head and substrate: (**a**) 1200 mm/min; (**b**) 1400 mm/min; (**c**) 1600 mm/min; (**d**) 1800 mm/min.

Figure 5 shows the morphology of the weld metal between the diamond saw blade head and the substrate at different welding speeds. As shown in Figure 5, with the increase in the welding speed, the shape of the cup-shaped melt pool gradually changes from wide to narrow. According to the heat input formula *Q* = *P*/*V*, it can be concluded that the heat input is inversely proportional to the welding speed [36,37]. A lower welding speed can extend the formation and solidification time of the welding melt pool, resulting in a larger melt pool and a wider weld metal formation, which may even lead to sputtering at the bottom of the welding joint, Figure 5a. 

**Figure 6:**
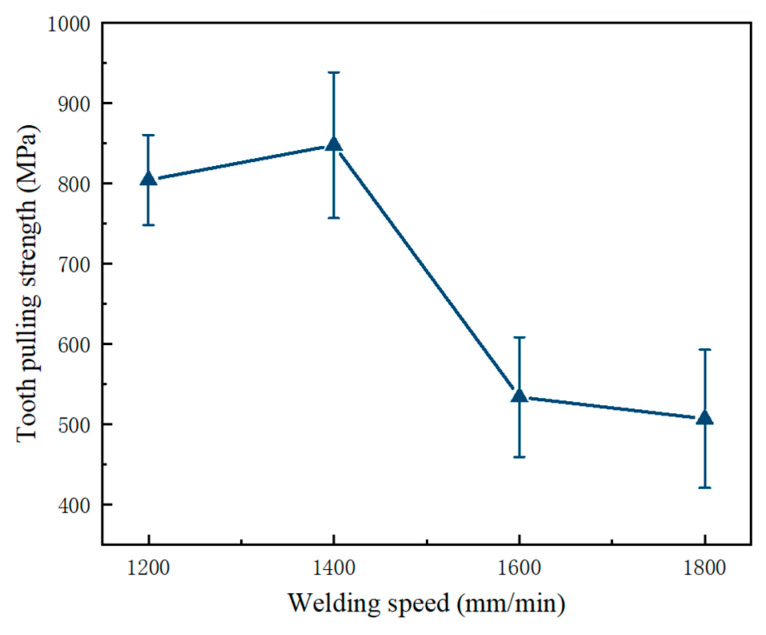
Effect of welding speeds on tooth-pulling strength of the welded joint between the diamond saw blade head and the substrate.

Figure 6 shows the tooth-pulling strength of the welded joint between the diamond saw blade and substrate at various welding speeds. The teeth’s strength initially increases with increasing welding speed, reaching a maximum value of 820 MPa at 1400 mm/min. Appropriate welding speed can produce a uniform and tight weld microstructure, improve tooth-pulling strength, and ensure the reliability of the welded joint. Conversely, excessive or insufficient welding speed can have a negative impact on the quality of the weld metal and decrease tooth-pulling strength. When the welding speed increases to 1600 mm/min, the tooth-pulling strength significantly decreases due to insufficient melting and solidification processes between the diamond saw blade and the substrate weld, resulting in uneven crystal structures and inclusions that finally decrease the toughness and strength of the weld metal. Too low a welding speed leads to excessive melting of the weld area and expansion of the heat-affected zone of the steel substrate, which results in defects such as joint porosity and cracks. Porosity, inclusions, and incomplete penetration defects in laser-welded diamond saw blades can negatively impact their quality and reliability by compromising structural integrity and reducing cutting performance. Mitigating these issues involves optimizing welding parameters and techniques, ensuring proper material preparation, and implementing rigorous quality control measures during manufacturing.

### 3.3. Microstructural Analysis

**Figure 7:**
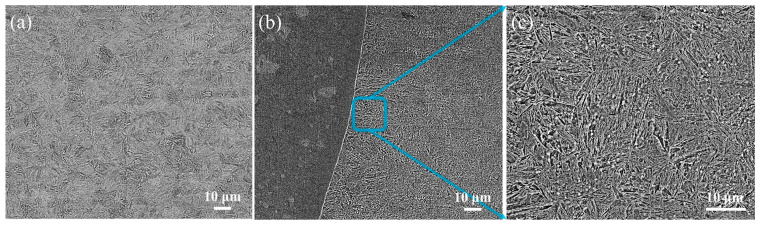
Metallographic microstructure: (**a**) microstructure of 30CrMo matrix; (**b**), (**c**) microstructure of heat-affected zone of 30CrMo matrix.

Figure 7 shows the microstructure of the 30CrMo matrix and the heat-affected zone of the matrix. The quenched and tempered martensitic microstructure in Figure 7a is formed in the 30CrMo matrix. Firstly, martensite is achieved through rapid cooling during the quenching processes. Then, tempered martensite is formed by the tempering processes. The quenched and tempered martensite microstructure possesses excellent toughness and resistance against brittleness. On the other hand, the heat-affected zone (Figure 7b,c) of the 30CrMo matrix shows a needle-like quenched martensitic microstructure. During welding, this region experiences high-temperature heating that surpasses the Ac3 line (austenite transformation line), resulting in the transformation of the steel. Subsequently, due to rapid cooling rates, a slender needle-like microstructure known as quenched needle-like martensite is formed. Because the microstructure typically exhibits high hardness, it also tends to be brittle. The significant coarsening observed in the microstructure closer to the melting zone is considered a key factor contributing to embrittlement within the heat-affected zone.

**Figure 8:**
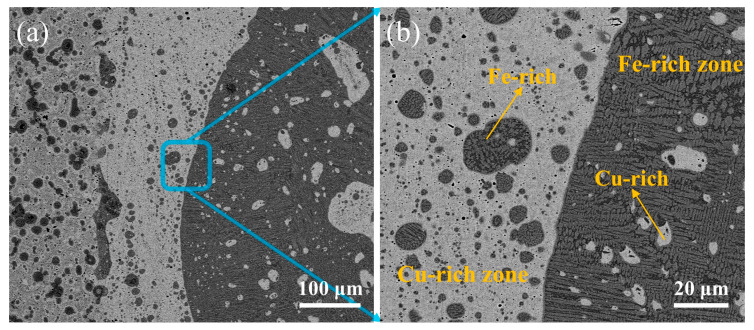
Microstructure of the molten zone of the weld seam: (**a**) SEM image; (**b**) enlarge image of (**a**).

Figure 8 shows the microstructure of the weld fusion zone under CBS. As illustrated, the weld metal is composed of the Cu-rich zone near the transition layer and the Fe-rich zone near the matrix, which are marked in Figure 8. Fe-rich spherical or dendritic segregation occurs in the Cu-rich zone. Cu-rich spherical segregation appears in the Fe-rich zone. This phenomenon arises due to the primary and secondary separation of the liquid phases during the laser welding of dissimilar metals such as Cu and Fe. Because neither intermetallic compounds nor solid solution can be formed between Cu and Fe in the weld metal, macroscopic segregation in the melting zone under high cooling rates and complex melting flow conditions is prone to be found in the laser welding of a Cu-based transition layer and a 30CrMo matrix. In addition, according to the Fe-Cu phase diagram, metastable mixed-phase gaps exist under subcooling conditions. Laser welding has the characteristics of rapid cooling and high undercooling. In addition, the melting point of the substrate is significantly higher than that of copper but its thermal conductivity is significantly lower than that of copper, which ultimately leads to the formation of two different phase regions in the weld metal.

**Figure 9:**
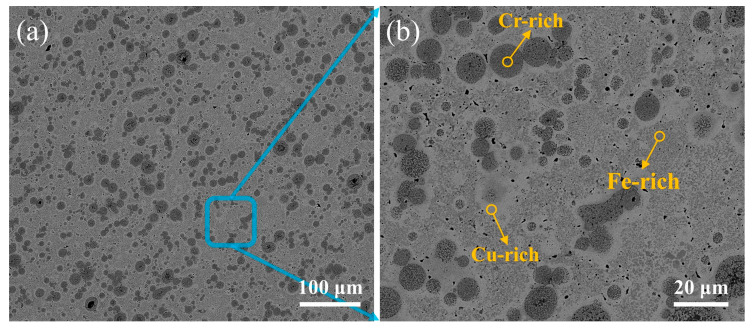
Microscopic structure of the transition layer of diamond saw blade tip: (**a**) SEM image; (**b**) enlarge image of (**a**).

Figure 9 shows the microstructure of the transition layer of the laser-welded diamond saw blade head. Through EDS energy spectrum analysis, the white area, the light gray area, and the dark gray spherical area in Figure 9 are identified as rich-Cu phase structures, rich-Fe phase structures, and rich-Cr phase structures, respectively. From the Fe Cu Ni and Fe Cu phase diagrams, it can be inferred that Cu and Fe elements are essentially insoluble in Ni, while Cu and Fe elements are completely immiscible. The Cu and Fe within the fusion zone of the welded joint undergo primary and secondary liquid phase segregation, resulting in the formation of Cu-rich regions exhibiting spherical or dendritic segregation with Fe enrichment, as well as Fe-rich regions exhibiting spherical segregation with Cu enrichment. With an increase in heat input, a gradual expansion of the Cu-rich regions was observed. Simultaneously, it was noted that the Cu-rich regions typically reside in the lower-left portion of the fusion zone, whereas the Fe-rich regions tend to be situated in the upper-right region. This phenomenon can be attributed to the higher density and lower melting point of Cu compared to Fe. During the laser-welding process, Cu solidifies first and, due to its greater weight, it occupies a lower position.

### 3.4. Phase Composition Analysis

**Figure 10:**
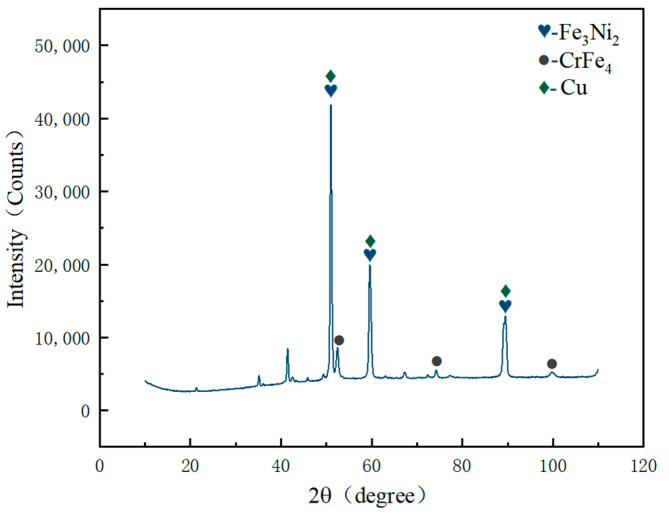
Micro-area XRD pattern of the molten zone in laser-welded joints.

According to the micro-XRD pattern of the laser-welded joint melting zone in Figure 10, it can be observed that the melting zone is composed of Cu solid solution phase and intermetallic compound phases such as Fe_3_Ni_2_ and CrFe_4_. Figure 10 depicts the phase distribution, revealing that the grains in the transition layer and molten zone (rich Cu area and rich Fe area) possess an FCC crystal structure. This outcome further confirms that the crystal structure of the Fe_3_Ni_2_ and CrFe_4_ phases detected in the micro-area XRD pattern is indeed an FCC structure. The presence of these compound phases results in solid solution strengthening on the welded joint, thereby enhancing blade tooth strength.

**Figure 11:**
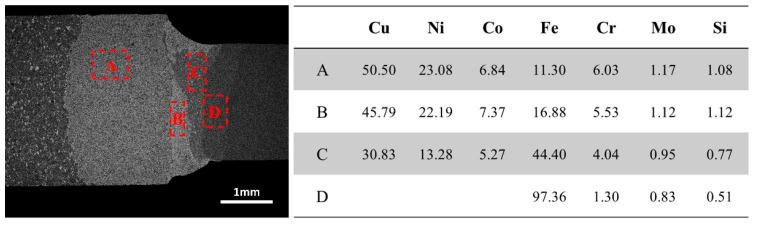
Distribution of elements on the welding joint surface between diamond saw blade and substrate.

In order to further analyze the elemental distribution in dissimilar welded joints, EDS analysis was conducted near the fusion lines on both sides and the results are illustrated in Figure 11. The transition layer of the blade primarily consists of Cu, Ni, Fe, Co, Cr, and Si elements. The molten zone includes Fe elements within the steel matrix as well as elements such as Cu, Ni, Co, and Cr from the transition layer. The heat-affected zone in the 30CrMo matrix is predominantly composed of Fe.

**Figure 12:**
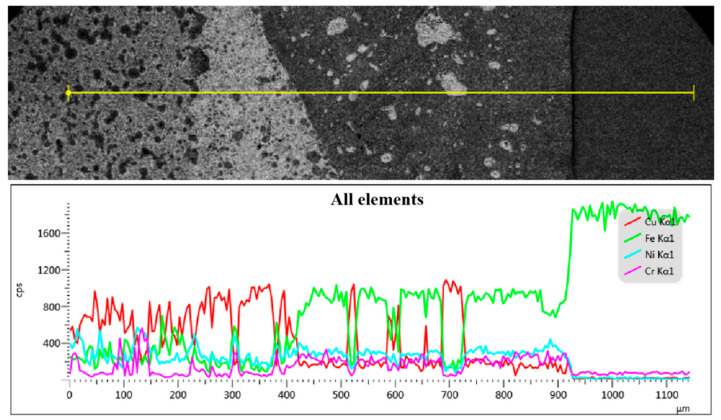
Line scanning distribution of main element composition in the welding joint between diamond saw blade and matrix.

Figure 12 illustrates the findings of the line scan analysis conducted along the transverse direction of the weld metal. The analysis reveals a gradual increase in the mass fraction of the Fe element from the transition layer and melting zone to the heat-affected zone. Conversely, the mass fractions of Cu, Ni, and Cr elements progressively decrease until they become negligible. The elements in the transition layer area appears disordered. Notably, the variation pattern of Ni and Cr elements on the transition layer side of the melting zone aligns with that of Fe, but contrasts with that of Cu. Additionally, in the melting zone near the substrate side, the mass fraction of Fe increases while that of Cu decreases. Remarkably, the heat-affected zone exhibits a significantly high mass fraction of Fe, reaching up to 98%.

### 3.5. EBSD Analysis

**Figure 13:**
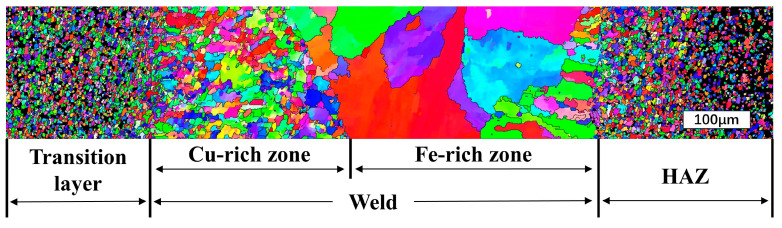
Inverse pole figure (IPF) image of the welding joint between the diamond saw blade and matrix.

**Figure 14:**
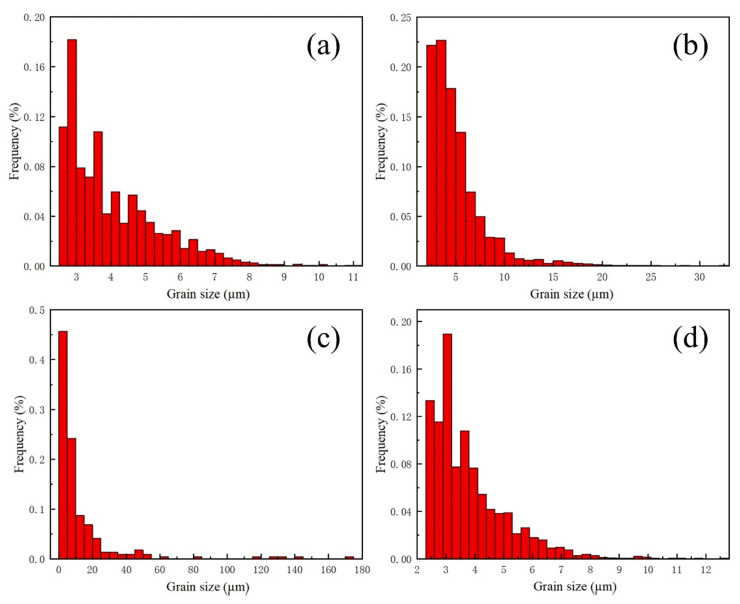
The grain size distribution of the welded joint between the diamond saw blade and matrix. (**a**) Grain size in transition layer; (**b**) grain size in melt zone side; (**c**) grain size in substrate side; (**d**) grain size in matrix heat-affected zone.

**Figure 15:**
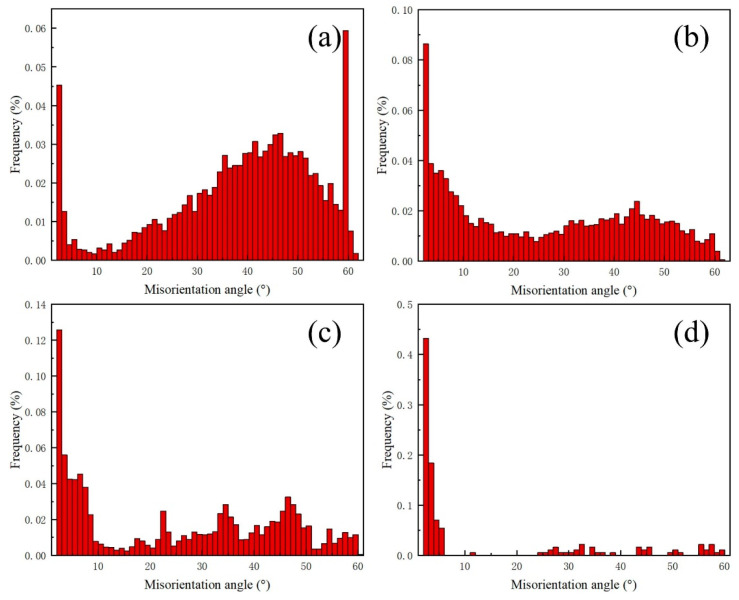
The misorientation angle distribution of the welded joint between the diamond saw blade and matrix. (**a**) Transition layer grain orientation angle; (**b**) melt zone side grain orientation angle; (**c**) melt zone side grain orientation angle; (**d**) matrix heat-affected zone grain orientation angle.

The grain size and orientation of laser-welded diamond saw blade joints were analyzed using EBSD [38,39], and the results are presented in Figure 13, Figure 14 and Figure 15. Figure 13 illustrates the IPF diagram of a 1600 w × 1400 mm/min welded joint. It can be observed from the figure that both the Cu-based transition layer area and the 30CrMo matrix area consist of fine grains, exhibiting a disordered color distribution and random grain orientation. The melting zone is divided into two regions by the boundary formed during the initial liquid phase separation of Fe and Cu. Due to the sintering of the Cu-based transition layer from pre-alloyed powder particles, it is evident that the grain size in the left Cu-rich zone is significantly smaller than that in the right Fe-rich zone. However, both zones exhibit larger grain sizes compared to those found in their respective base materials. The melting zone exhibits columnar dendrites, which grow in the opposite direction of the temperature gradient and lack a distinct preferred orientation. This columnar crystal structure is likely attributed to the rapid cooling rate and high undercooling level during laser welding.

Figure 14a–d show the grain size distribution of the Cu-based transition layer zone, melting zone (transition layer side and substrate side), and 30CrMo substrate zone, respectively. Notably, the grain size within the molten zone is larger than that of the base metal, with average sizes of 4.03 µm and 3.96 µm, respectively. Furthermore, the average size of the transition layer and substrate sides in the melting zone is 5.36 µm and 13.78 µm, with even large grains of 148.56 µm. Figure 15a–d show the distribution of grain orientation angle differences in the Cu-based transition layer zone, melting zone (transition layer side and substrate side), and 30CrMo substrate zone, respectively. Many large-angle grain boundaries are present in the weld metal, and numerous twinnings are observed on both sides. However, no twinning is found in the center of the melting zone.

### 3.6. Microhardness Distribution

**Figure 16:**
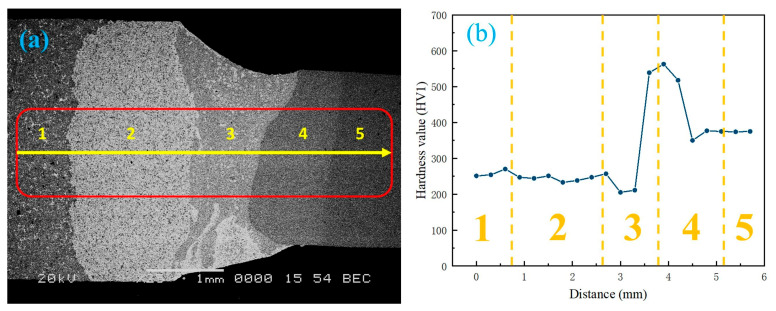
Microhardness of laser-welded diamond saw blade weld seam: (**a**) measurement points; (**b**) microhardness distribution.

Figure 16 shows the microhardness changes at the transverse corresponding positions (1~5) of the laser-welded saw blade weld metal. Regions 1 and 2 represent the working layer and transition layer of the cutting head, respectively. The hardness variations in these two areas are relatively minimal, with an average hardness of 256 HV and 241 HV, respectively, maintaining the same level as that of the working layer and transition layer of the hot-pressed sintered blade prior to welding. The microhardness in the melting zone (Zone 3) significantly decreased, reaching 211 HV. Extending from this melting zone to the matrix heat-affected zone with a width of approximately 800 μm reveals a sharp increase in microhardness from 200 HV to 550 HV. This increase is attributed to higher Fe content along with lower Cu and Ni content within this range, exhibiting characteristics similar to those found in as-cast columnar crystals displaying inverse compositional changes. The relative decrease in hardness from 500 HV in the heat-affected zone of the matrix (region 4) to 380 HV in the matrix (region 5) further confirms the existence and characteristics of quenched needle-like martensite. In a welded joint, the fusion zone typically undergoes rapid thermal cycling, resulting in elevated cooling rates and the formation of a refined microstructure. This accelerated cooling engenders heightened hardness within the fusion zone in comparison to the base metal and heat-affected zone (HAZ). Consequently, a brittleness disparity arises, rendering the fusion zone more susceptible to fracturing. Under applied loads, stress distribution across the joint is non-uniform. The region characterized by increased hardness (the fusion zone) tends to exhibit greater stiffness and reduced ductility. Conversely, the region with diminished hardness (the HAZ) may manifest enhanced ductility. The presence of varying microhardness levels at the interface can also induce stress concentration, thereby precipitating potential failure.

### 3.7. Analysis of Fracture Morphology

**Figure 17:**
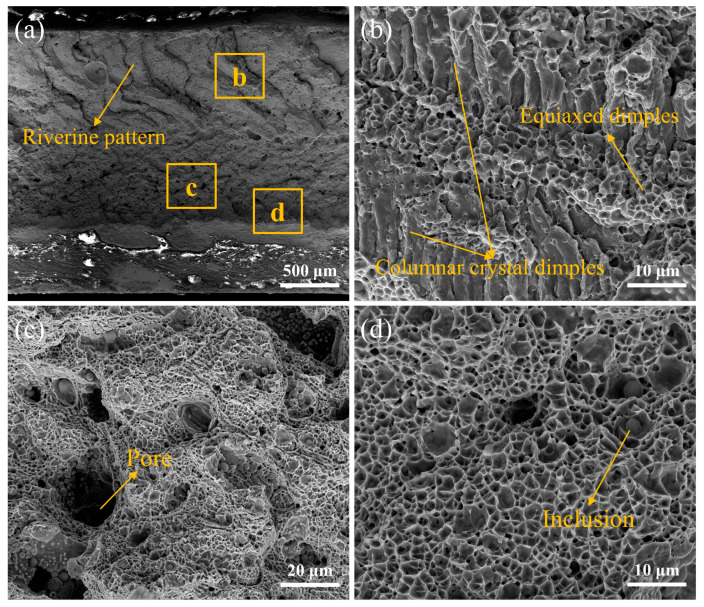
Fracture morphology of diamond saw blade head in laser welding: (**a**) fracture morphology, (**b**) enlarge image of region b; (**c**) enlarge image of region c; (**d**) enlarge image of region d.

After multiple indentation tests, it was observed that fractures predominantly occurred at the interface between the fusion zone and the heat-affected zone. This phenomenon can be visually examined from the cross-sectional observation of the joint post-indentation, as depicted in Figure 17, where inclusions are indicated. From Figure 17a, it can be seen that the fracture surface exhibits a complex morphology, resembling a mixed fracture with significant plastic deformation during the fracturing process. 

The fracture surface is composed of dimple and local cleavage facets. By referring to the schematic diagram of trigger teeth, it becomes evident that shear stress is initially experienced by the cutting head, resulting in a river-like pattern at the upper end of the sample. As tooth pulling progresses, tensile stress occurs in the middle region of the blade and equiaxed dimples are formed. Finally, at the lower end, shear stress causes fracture with shallow dimples. Figure 17b shows the fracture feature of columnar crystal dimples at the upper and lower ends, and the fracture feature of equiaxed dimples in the middle. In Figure 17c, many pores are produced, which may be due to the presence of phases with lower melting points in the transition layer during laser welding. These pores cause stress concentration within welded joints and consequently decrease their strength. A number of criteria, including adequate joint design, effective shielding gases, and careful selection of welding parameters (e.g., laser power, travel speed, and focal location), should be taken into account in order to prevent porosity in laser welds and achieve the requisite stability. In order to reduce porosity formation, it is also critical to minimize impurities, maintain constant welding conditions, and ensure clean and adequately prepared surfaces. Furthermore, numerous inclusions within ductile dimples also affect welding strength.

**Figure 18:**
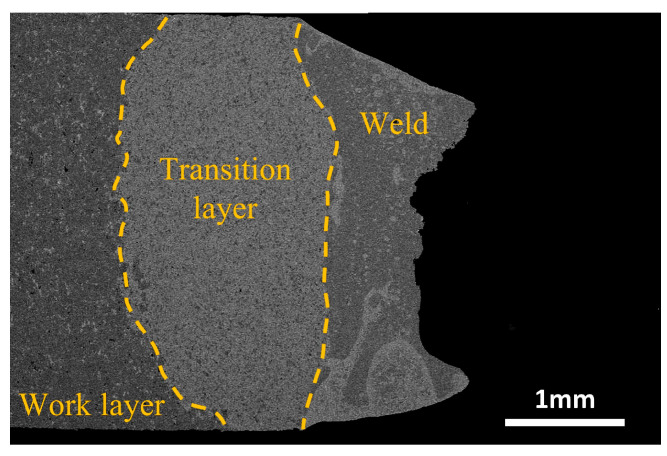
Cross-section of laser-welded diamond saw blade head fracture.

The cross-sectional view of the fracture surface of the laser-welded diamond saw blade after tooth pulling is depicted in Figure 18. The fracture site is located at the interface between the melting zone and the heat-affected zone of the substrate, which is consistent with the results of our previous microhardness analysis. This phenomenon can be attributed to quenched needle-like martensite with high strength and poor brittleness during the rapid cooling of laser welding. EDS analysis reveals a small amount of Cu element present in the heat-affected zone of the matrix, which tends to accumulate at grain boundaries and diminishes bonding strength among Fe grain boundaries, ultimately leading to stress concentration. It should be noted that, under welding process parameters (1600 W, 1400 mm/min), fractures occur within the heat-affected zone of the substrate for cutting heads and these fractures exhibit the inclusion of substrate material on their surfaces, further confirming the strong joining strength of the molten zones under this specific welding process.

## 4. Conclusions

(1) The laser-welded diamond saw blades with a tooth-pulling strength reaching as high as 819 MPa are obtained at the optimized welding parameters with a laser power of 1600 W and a welding speed of 1400 mm/min. However, during the process of laser welding, it is essential to carefully adjust the heat input. Too low a heat input may result in incomplete penetration defects, while excessively higher heat input could lead to issues such as spattering, splashing, porosity, and cracks.
(2) The cross-sectional morphology of laser-welded diamond saw blades can be divided into the transition layer of the base material, the welding zone (including Cu-rich and Fe-rich regions), the matrix heat-affected zones, and the base material zones. In the welding zone, due to multiple segregations of Fe and Cu under supercooling conditions, spherical or dendritic iron segregation near the copper matrix and spherical copper segregation near the 30CrMo matrix are formed. Simultaneously, large-sized grains with dimensions reaching 148.56 µm are present. The 30CrMo matrix heat-affected zone exhibits coarse needle-like martensitic microstructures from quenching. The 30CrMo matrix is composed of quenching and tempering martensite.
(3) The microhardness of the welded joint sharply increases at the boundary between the fusion zone and the heat-affected zone of the base metal, reaching a maximum of 550 HV. Through the tooth-pulling strength test, it was observed that fractures typically occur at the interface between the laser-welded fusion zone and the heat-affected zone of the base metal. This phenomenon is primarily attributed to the rapid cooling during laser welding, leading to the formation of high-strength but brittle quenched needle-like martensite. The requirements for the shape of the diamond saw blade result in a thicker cutting edge compared to the base metal, causing stress concentration at the interface and further increasing the risk of fracture.

## Figures and Tables

**Table 1:** Chemical component of transition layer.

Element	Cu	Ni	Fe	Co	Cr
wt%	58.6	17.2	11.8	7.3	5.1

**Table 2:** Chemical component of 30CrMo matrix.

Element	C	Si	Mn	P	Cr	Mo	Fe
wt%	0.321	0.352	0.487	0.018	1.082	0.245	Bal.

**Table 3:** Welding parameters joining the diamond cutting head and 30CrMo matrix.

No.	1#	2#	3#	4#	5#	6#	7#	8#
Welding power (w)	1600	1600	1600	1600	1400	1500	1600	1700
Welding speed (mm/min)	1200	1400	1600	1800	1400	1400	1400	1400

## Data Availability

All data supporting the conclusions of this manuscript are included within the manuscript.

## References

[1] Vieira V.F., Shigaki Y., Martins P.S., Ba E.C.T., Dias C.A.R. (2023). Dias, Nanoindentation test of a DLC coated high-speed steel substrate using a two-dimensional axisymmetric finite element method. Diam. Relat. Mater..

[2] Pan R., Yang D., Zhou T., Feng Y., Dong Z., Yan Z., Li P., Yang J., Chen S. (2023). Micro-welding of sapphire and metal by femtosecond laser. Ceram. Int..

[3] Kong X., Su Z., He T., Wu J., Wu D., Zhang S. (2022). Development and properties evaluation of diamond-containing metal composites for fused filament fabrica-tion of diamond tool. Diam. Relat. Mater..

[4] Wang L., Guo S., Gao J., Yang L., Hu T., Peng J., Hou M., Jiang C. (2017). Microwave sintering behavior of FeCuCo based metallic powder for diamond alloy tool bit. J. Alloys Compd..

[5] Tao Y., Gan J., Sun W., Zhou Y., Duan L., Wen S., Shi Y. (2023). High-strength and wear-resistant Co-Cr-Mo/diamond composites fabricated by selective laser melting. Int. J. Refract. Met. Hard Mater..

[6] Hou M., Gao J., Yang L., Ullah E., Hu T., Guo S., Hu L., Li Y. (2020). The role of pre-alloyed powder combined with pressure-less microwave sintering on performance of super-hard materials. J. Alloys Compd..

[7] Li W., Zhang J. (2021). Microstructure and mechanical properties of a novel Cu-Fe based matrix for diamond segments. Proceedings of the 2011 International Conference on Remote Sensing, Environment and Transportation Engineering.

[8] Hou M., Guo S., Yang L., Gao J., Peng J., Hu T., Wang L., Ye X. (2018). Fabrication of Fe–Cu matrix diamond composite by microwave hot pressing sintering. Powder Technol..

[9] Sung J.C., Sung M. (2009). The brazing of diamond. Int. J. Refract. Met. Hard Mater..

[10] Konstanty J. (2013). Sintered diamond tools: Trends, challenges and prospects. Powder Metall..

[11] Aminzadeh A., Parvizi A., Safdarian R., Rahmatabadi D. (2020). Comparison between laser beam and gas tungsten arc tailored welded blanks via deep drawing. Proc. Inst. Mech. Eng. Part B J. Eng. Manuf..

[12] Konstanty J. (2011). Powder Metallurgy Diamond Tools.

[13] Zhang L. (2021). Filler metals, brazing processing and reliability for diamond tools brazing: A review. J. Manuf. Process..

[14] Zhang S., To S., Zhang G. (2017). Diamond tool wear in ultra-precision machining. Int. J. Adv. Manuf. Technol..

[15] Hsieh Y.-Z., Lin S.-T. (2001). Diamond tool bits with iron alloys as the binding matrices. Mater. Chem. Phys..

[16] Yao C., Xu B., Zhang X., Huang J., Fu J., Wu Y. (2009). Interface microstructure and mechanical properties of laser welding copper–steel dissimilar joint. Opt. Lasers Eng..

[17] Costa A.P., Quintino L., Greitmann M. (2003). Laser beam welding hard metals to steel. J. Mater. Process. Technol..

[18] Norouzian M., Amne Elahi M., Plapper P. (2023). A review: Suppression of the solidification cracks in the laser welding pro-cess by controlling the grain structure and chemical compositions. J. Adv. Join. Process..

[19] Ikawa N., Shimada S., Tsuwa H. (1982). Microfracture of diamond as fine tool material. CIRP Ann..

[20] Guihong Y. (2018). Effect mechanism of formula pre-alloying on improving hot-pressed diamond bit iron-rich matrix performance. Geol. Sci. Technol. Inf..

[21] Li T., Xu J., Bi X., Li R. (2022). Microstructure evolution and crack propagation mechanism during laser lap welding of Ti6Al4V and DP780 steel with CoCrNi powder. Mater. Des..

[22] Xin J., Zhang H., Sun W., Huang C., Wang S., Wei J., Wang W., Fang Z., Wu D., Li L. (2021). The microstructures and mechanical properties of dissimilar laser welding of copper and 316L stainless steel with Ni interlayer. Cryogenics.

[23] Li H., Sun D., Gu X., Dong P., Lv Z. (2013). Effects of the thickness of Cu filler metal on the microstructure and properties of laser-welded TiNi alloy and stainless steel joint. Mater. Des..

[24] Li H., Sun D., Cai X., Dong P., Wang W. (2012). Laser welding of TiNi shape memory alloy and stainless steel using Ni interlayer. Mater. Des..

[25] Wei Z., Najafi A., Taheri M., Soleymani F., Didehvar N., Khalaj G. (2023). The Effect of an Ultrasonic Field on the Microstructure and Tribological Behavior of ZrB_2_/ZrC+Ni60A/WC Composite Coating Applied by Laser Cladding. Coatings.

[26] Khorrami M.S., Mostafaei M.A., Pouraliakbar H., Kokabi A.H. (2014). Study on microstructure and mechanical characteristics of low-carbon steel and ferritic stainless steel joints. Mater. Sci. Eng. A.

[27] Dhokey N.B., Utpat K., Gosavi A., Dhoka P. (2013). Hot-press sintering temperature response of diamond cutting tools and its correlation with wear mecha-nism. Int. J. Refract. Met. Hard Mater..

[28] Kenéz A.Z., Bagyinszki G. (2018). Investigation of Laser Welding Technology of Diamond Drilling Segments. Acta Mater. Transylvanica.

[29] Abroug F., Monnier A., Arnaud L., Balcaen Y., Dalverny O. (2022). High cycle fatigue strength of additively manufactured AISI 316L Stainless Steel parts joined by laser welding. Eng. Fract. Mech..

[30] Ghosh P.S., Sen A., Chattopadhyaya S., Sharma S., Singh J., Li C., Królczyk G., Rajkumar S. (2022). Progressive developments and challenges in dissimilar laser welding of steel to various other light alloys (Al/Ti/Mg): A comprehensive review. Heliyon.

[31] Ogawa Y., Horita T., Iwatani N., Kadoi K., Shiozawa D., Sakagami T. (2022). Evaluation of fatigue strength based on dissipated energy for laser welds. Infrared Phys. Technol..

[32] Idriss M., Mirakhorli F., Desrochers A., Maslouhi A. (2023). Fatigue behaviour of AA5052-H36 laser-welded overlap joints: Effect of stitch-weld orientation and gap bridging. Int. J. Fatigue.

[33] Gu H., Väistö T., Wei C., Li L., Ren X., Qian L. (2023). A coupled ray-tracing based CFD and cellular automaton model for predicting molten pool formation and microstructure evolution in narrow gap laser welding. Int. J. Heat Mass Transf..

[34] Satbhai O., Neog S.P., Karagadde S., Samajdar I., Jaya B.N., Kumar H., Ravikumar R., Mythili R., Ghosh C., Dasgupta A. (2023). A novel macroscopic computational methodology to predict the locations and orientation of solidification-cracks: Application to pulsed laser welding. Int. J. Heat Mass Transf..

[35] Sirohi S., Pandey S.M., Tiwari V., Bhatt D., Fydrych D., Pandey C. (2023). Impact of laser beam welding on mechanical behaviour of 2.25Cr–1Mo (P22) steel. Int. J. Press. Vessel. Pip..

[36] Horník P., Šebestová H., Novotný J., Mrňa L. (2022). Laser beam oscillation strategy for weld geometry variation. J. Manuf. Process..

[37] Jabeen R., Cosson B., Asséko A.C.A., Verstraete S., Desplentere F., Park C.H. (2023). Effect of fibre orientation on the light scattering during laser transmission welding. J. Manuf. Process..

[38] Rasouli A., Naffakh-Moosavy H. (2023). The effect of Nd:YAG laser pulse duration and post-weld heat treatment on the microstructure and mechanical properties of laser-welded NiTi shape memory alloy. J. Mater. Res. Technol..

[39] Vâlsan D.-D., Bolocan V., Burcă M., Crăciunescu C.-M. (2023). Experiments on Nd:YAG pulsed laser welding of thin sheets. Mater. Today Proc..

